# Analysis of the Gut Microbiome of Rural and Urban Healthy Indians Living in Sea Level and High Altitude Areas

**DOI:** 10.1038/s41598-018-28550-3

**Published:** 2018-07-04

**Authors:** Bhabatosh Das, Tarini Shankar Ghosh, Saurabh Kedia, Ritika Rampal, Shruti Saxena, Satyabrata Bag, Ridhima Mitra, Mayanka Dayal, Ojasvi Mehta, A. Surendranath, Simon P. L. Travis, Prabhanshu Tripathi, G. Balakrish Nair, Vineet Ahuja

**Affiliations:** 1 Molecular Genetics Laboratory, Centre for Human Microbial Ecology, Translational Health Science and Technology Institute, NCR Biotech Science Cluster, Faridabad, 121001 India; 2 Department of Gastroenterology and Human Nutrition, All India Institute of Medical Sciences, New Delhi, India; 3 Translational Gastroenterology Unit, Oxford University Hospitals, Oxford, UK; 4 Present Address: Research Policy and Cooperation Unit, Communicable Diseases Department, World Health Organization (WHO), Mahatma Gandhi Marg, Indraprastha Estate, New Delhi, 110 002 India

## Abstract

The diversity and basic functional attributes of the gut microbiome of healthy Indians is not well understood. This study investigated the gut microbiome of three Indian communities: individuals residing in rural and urban (n = 49) sea level Ballabhgarh areas and in rural high altitude areas of Leh, Ladakh in North India (n = 35). Our study revealed that the gut microbiome of Indian communities is dominated by *Firmicutes* followed by *Bacteroidetes*, *Actinobateria* and *Proteobacteria*. Although, 54 core bacterial genera were detected across the three distinct communities, the gut bacterial composition displayed specific signatures and was observed to be influenced by the topographical location and dietary intake of the individuals. The gut microbiome of individuals living in Leh was observed to be significantly similar with a high representation of *Bacteroidetes* and low abundance of *Proteobacteria*. In contrast, the gut microbiome of individuals living in Ballabhgarh areas harbored higher number of *Firmicutes* and *Proteobacteria* and is enriched with microbial xenobiotic degradation pathways. The rural community residing in sea level Ballabhgarh areas has unique microbiome characterized not only by a higher diversity, but also a higher degree of interindividual homogeneity.

## Introduction

**Figure 1:**
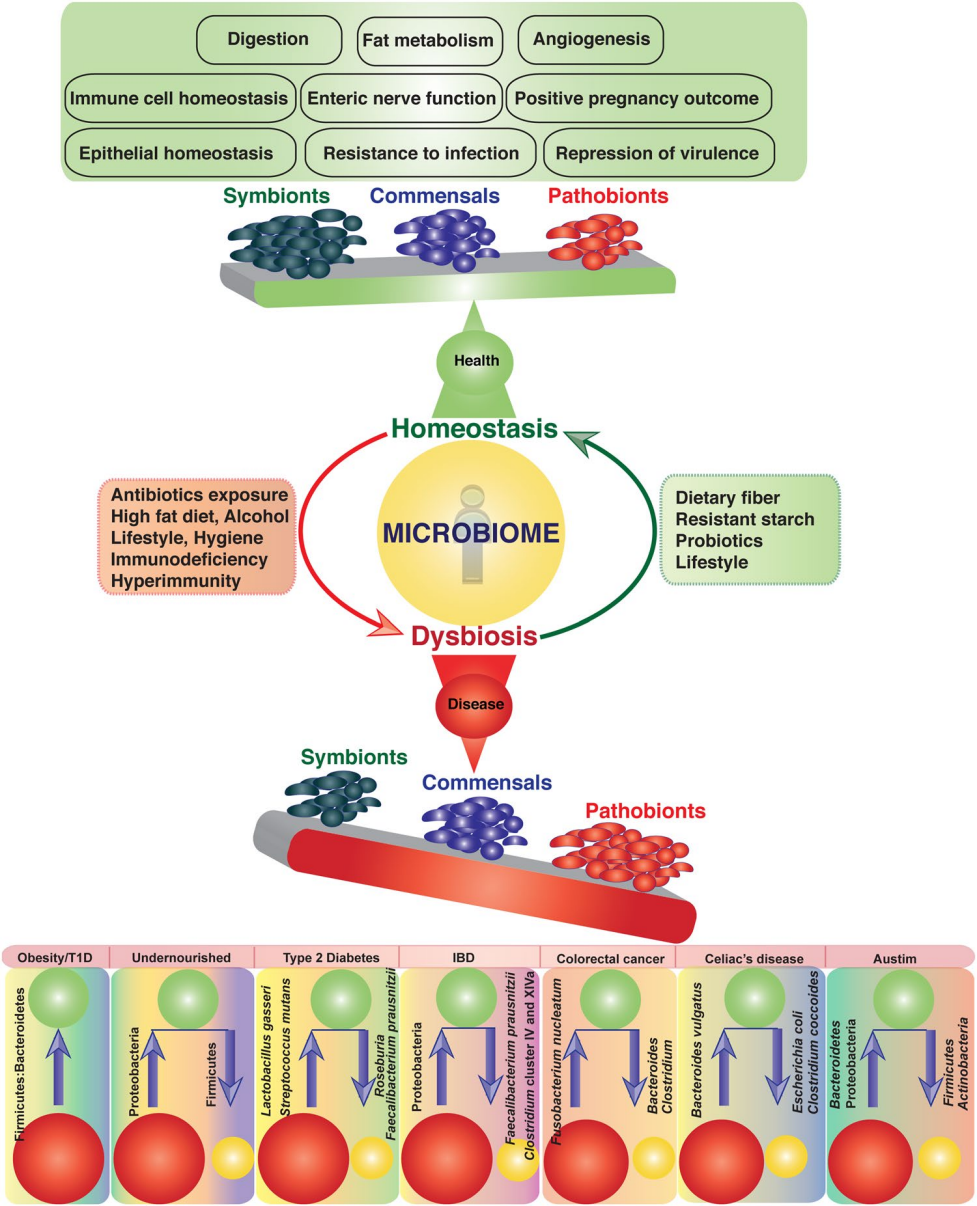
Role of the gut microbiome in health and diseases. Several factors including diet, antibiotics, host genetics etc. can influence the microbial community structure in the gut and lead to dysbiosis that may induce inflammation and health disorders like IBD, malnutrition, colorectal cancer and several others.

The human gastrointestinal tract (GIT), the major site of nutrient assimilation and micronutrient production, is populated with trillions of microbial cells from all the three domains of life (*Archaea*, *Bacteria* and *Eukarya*). Bacterial species, in particular, play a crucial role in the digestion of complex dietary polysaccharides by providing several enzymatic functions that are not encoded in the human genome^1^. Several species of bacteria metabolize bile salts and repress the virulence of enteric pathogens, transform pro-drugs into active drugs and reduce the toxicity of xenobiotic compounds by chemical transformation^2,3^. In addition, the gut microbiota plays an important role in the synthesis of vitamins, neurotransmitters and other metabolites, which are the key components of human health and can modulate host immunity, cytokine production, development of gut-associated lymphoid tissues (GALTs) and development and maturation of gut-specific immune system^4–6^. Healthy immune system and balanced community of the gut microbiota are crucial for human health. Abrupt changes in microbiota (dysbiosis) can potentiate several health disorders including malnourishment, inflammatory bowel disease (IBD), metabolic and neuronal diseases, colorectal cancer, coronary heart disease, rheumatoid arthritis, autoimmune and psychiatric disorders (Fig. 1).

Microbes living in the GIT of healthy individuals across the globe tend to adopt distinct community structures, called enterotypes^7^. People living in different continents have different intestinal microbiota and several factors including diet, environment, antibiotic usages and host genetics could play important role in shaping community structure of the gut microbiota (Fig. 1)^8,9^. India, the second highest populated country on the planet, houses more than 2000 major human communities living in approximately 1900 territorial units with well distinct geography^10^. However, in spite of this immense variety, our knowledge on the microbial diversity and genetic makeup of the microbiota inhabiting the gut of healthy adult Indians is limited. Few studies have investigated the gut microbiome of Indian subjects, but the outcomes of such studies are not comprehensive and restricted due to several factors like limited sample size, inefficient and inconsistent community DNA extraction methods, and variations in the sequencing technologies used to analyze the gut microbiomes^11–13^. Considering the distinct environment, various food habits and unique socio-economic culture, we anticipated that microbiome research on Indian subjects, investigating specific hitherto unexplored sub-populations, has unique opportunity. In this study, we analyzed the fecal microbiome of 84 healthy adult Indians living in rural and urban areas of different parts of the country. Our study indicated that the microbial populations in the gut of healthy Indians are dominated by *Firmicutes* followed by *Bacteroidetes*, *Actinobacteria*, *Proteobacteria*, *Spirochetes*, *Verrucomicrobia* and *Fusobacteria*. Findings of the present study will help to understand the gut microbial diversity of the healthy Indians living in different parts of the country and the factors that influence microbial diversity and their contribution to normal human physiology.

## Methods

### Geographical Regions

The sea level rural and urban sites comprised 29 villages and 27 urban wards in Ballabhgarh block in Faridabad district of Haryana state, which is 40 kilometers from Delhi, the capital of India. It lies between 28°10′ and 28°29′ north latitude and 77°06′ to 77°33′ east longitude and 228 m (748 ft) above sea level. High altitude samples were taken from individuals living in Leh, which is a district of Ladakh, the highest plateau of the Indian state of Jammu and Kashmir with a height of 3,500 m (11,500 ft.) above sea level. Leh is located between the Kunlun mountain range in the north and the main Great Himalayas to the south. It lies between 32° and 36° north latitude and 75° to 80° east longitude.

### Subject recruitment and sample collection

Fecal samples were collected from healthy relatives accompanying patients to primary health Centre or district health Centre. Healthy adults were recruited if they were between 18–60 years of age and if they reported negative for the following items: (i) any history of concurrent acute medical illness (ii) any symptoms pertaining to gastrointestinal (GI) disease (nausea/vomiting/pain in abdomen/diarrhea/blood in stool) (iii) any history of chronic illness (diabetes/hypertension/heart disease/kidney disease/liver disease/malignancy) (iv) any history in the past 3 months of intake of antibiotics/antifungals/antivirals/painkillers (v) any history of high risk sexual behavior (vi) any history of consumption of illicit drugs within preceding six months (vii) any history of chronic GI diseases (IBD/inflammatory bowel syndrome (IBS)/chronic constipation/chronic diarrhea/abdominal tuberculosis) (viii) any history of GI malignancy or strong family history of colorectal cancer (ix) any history of other chronic illnesses such as autoimmune disease (multiple sclerosis/connective tissue disorders) or atopic disease (moderate–severe asthma, eczema, eosinophilic disorders of the GIT), metabolic syndrome, obesity or moderate to severe undernutrition/malnutrition. In addition, stool test for ova/cyst and stool culture test was also done.

The details of 84 subjects recruited in the present study are provided in the Supplementary Table S1. People living in the Leh regions are the descendants of a mixed race of Mons of North India, Mongols of Central Asia and Dards of Baltistan. People living in the Ballabhgarh urban and rural regions are mostly Aryan descendants. Participants who provided informed consent were included in the present study. The study was approved by the All India Institute of Medical Sciences, New Delhi ethics committee (IEC/NP-28/09.01.2015,OP-2/01.04.2016). Recombinant DNA studies were performed in “accordance” with the approved guidelines of Translational Health Science and Technology Institute (THSTI) biosafety committee. All other experimental protocols used in this study were carried out in “accordance” with the relevant guidelines and standard operating procedure (SOP) of Centre for Human Microbial Ecology (CHME).

### DNA extraction and pyro-sequencing

The fecal samples were kept at −80°C before extraction of genomic DNA. Around 200 mg frozen samples were used for DNA extraction using THSTI method^14^. The quality and quantity of DNA were assessed using Biospectrometer (Eppendorf, Germany) and 0.8% agarose gel electrophoresis. Variable regions V1-V5 of the 16S rRNA genes were amplified in 50 μl reaction volume using 0.1 ng of template DNA and 27 F(C1) and 926 R(C5) primers. The 950-bp long PCR products were gel purified using QIAquick gel elution Kit (Qiagen, Germany). Equimolar concentration amplicon libraries were mixed and sequenced by using Roche 454 GS FLX+ pyrosequencer at THSTI, India. Whole genome sequencing of the gut microbial genomic DNA was done as described previously^13^. Sequence reads obtained in FASTQ format were evaluated by FASTQC (http://www.bioinformatics.babraham.ac.uk/projects/fastqc/), using default parameters.

### Data processing, OTU clustering and taxonomic profiling

The samples were quality filtered and demultiplexed using the Next Generation Sequencing (NGS) tag cleaner software^15^. Operational Taxonomic Units (OTUs) were predicted by clustering the sequences with identities greater than 97% using UCLUST software package^16^. The genus and phylum affiliations of the representative sequences corresponding to each OTU were predicted using the Naïve-Bayesian based RDP classifier^17^. The species level affiliations of the representative sequences were obtained by performing a BLAST search of the representative sequences with an in-house version SILVA database^18^.

Based on the taxonomic affiliations of the representative sequences, the number of sequences belonging to each taxonomic group was then cumulated. Abundances of the various taxa in a given sample were calculated as the total number of sequences assigned to a given taxa divided by the total number of sequences in that sample. Scaled abundances (with values 0 to 1) were obtained by comparing the abundances of each taxa across samples. Across the samples, the maximum and the minimum abundances of each taxon was obtained and assigned as 1 and 0, respectively. For the other samples, the scaled abundances as

$${\rm{Abundance}}-{\rm{Minimum}}\,{\rm{Abundance}}/{\rm{Maximum}}\,{\rm{Abundance}}-{\rm{Minimum}}\,{\rm{Abundance}}$$

### Inferred functional profiling

In order to identify the differentially abundant functional processes and pathways across the three cohorts, predictive functional profiling was performed on the taxonomic profiles obtained using the 16S rRNA gene amplicon sequencing approach using the PICRUSt method^19^. In order to test the reliability of these predictions, we sequenced a subset of gut microbiomes using the shotgun metagenomic approach and performed the profiling of the functional processes and pathways using the HUMAnN method^20^ and compared the same with the PICRUSt based predictions for the corresponding microbiomes.

### Statistical analysis

Group wise comparisons of abundance of various taxa in the different cohorts were performed using Kruskal-Wallis H test and Mann-Whitney U tests (for pairwise comparisons). Multiple test corrections were performed using Benjamini-Hochberg method of R. Power calculations of the statistical comparisons were obtained using the G*Power program^21^. The method adopts the Asymptotic Relative Efficiency (ARE) approach to compute the ideal sample size of the groups compared in the empirical statistical comparison tests. The power analysis identified that with the current sample sizes (35, 25, 24), the comparisons could still identify groups (with P-value < 0.05) with a power of greater than 0.75. Random Forest classifications were performed using the ‘randomForest’ package of the R. PERMANOVA and ANOSIM were performed respectively using the ‘adonis’ and ‘anosim’ programs implemented in the ‘vegan’ package of R. Weighted unifrac based distance measures were obtained using the GUniFrac package of R, by providing the OTU abundance profiles (across samples) and the mutual phylogenetic tree of the OTUs as input. The tree containing the mutual relatedness of the OTUs was generated using the make_phylogeny.py program, implemented in the QIIME package^22^, after aligning the OTU representative sequences on the template of the GreenGenes taxonomy database (v13)^23^.

## Results

### Study population and dietary information and Sequencing outputs

A total of 84 adult healthy subjects were included in the present study. Leh group consisted of 35 adults (20 male) with a mean age of 35.5 ± 10.6 years (range: 18–59 years); Ballabhgarh rural had 25 adults (12 male) with a mean age of 34 ± 7.1 years (range: 22–49 years) and Ballabhgarh urban cohort included 24 adults (13 male) with a mean age of 36 ± 8.1 years (range: 24–58 years). The dietary habits of the subjects were vegetarians, non-vegetarians and eggetarians (Suppl. Table S2). Power analysis indicated that empirical comparisons on the given sample sizes could still identify markers with effect size of greater than 0.7 and P-value (alpha) <0.05 with a power of greater than 0.75. Self-collected fecal samples were characterized to reveal the composition of the microbiota and their genomic repertoires. The amplicon sequencing produced a total of 1,815,423 processed reads (post quality filtering and de-multiplexing), with an average 20,400 reads per subjects (average varying between 14605 and 26132 reads across cohorts). The average read length obtained 616 bps, which cover V3-V5 regions of 16S rRNA gene (cohort averages ranging from 604 to 686 bps) (Table 1; Suppl. Table S1). Within the cohorts, the average number of OTUs and species level Simpson Diversity varied from 1212–1685 and 0.65 to 0.74, respectively (Table 1).

**Table 1:** Sample summary showing the variation of per-sample pyrosequencing data and the age of the subjects within each cohort.

Cohort	Age (yrs)	Average Sequence Length (bp)	Reads per sample	Number of OTUs* per sample	Species Level Sample Simpson Diversity
Leh	35.7 ± 1.8	604.9 ± 1.4	16157 ± 1494	1352 ± 154	0.65 ± 0.01
Ballabhgarh Urban	34.0 ± 1.4	645.9 ± 9.1	28858 ± 6832	1212 ± 53	0.74 ± 0.01
Ballabhgarh Rural	36.0 ± 1.7	686.4 ± 4.7	21789 ± 481	1685 ± 384	0.73 ± 0.02

^*^Non Singleton OTUs.

### Taxonomic Composition and Diversity of Fecal Microbiota

**Figure 2:**
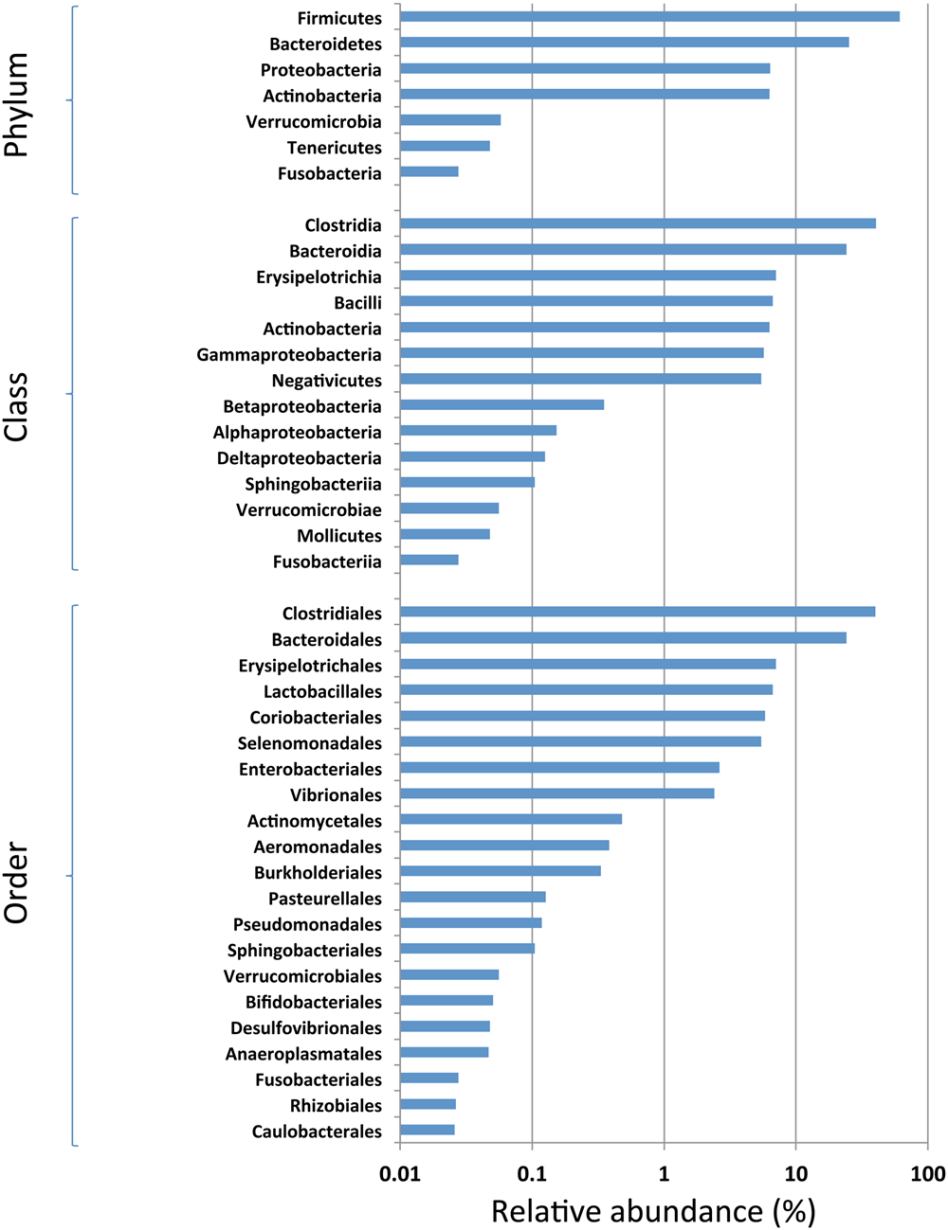
Overall composition of the gut microbiome of Indian subjects. The gut microbiota of the Indians are dominated by *Firmicutes* (62%), followed by *Bacteroidetes* (24%), *Actinobacteria* (5.2%) and *Proteobacteria* (4.2%). Members of the Verrucomicrobia, Tenericutes and Fusobacteriaare also present in the gut of most of the study subjects although, their abundance is low.

The taxonomic composition of the fecal microbiomes was then investigated at the levels of phylum, class, order, genus and species. The sequencing depth employed here revealed a total of 228 bacterial genera and 9 different phyla among all the 84 subjects. Overall, the fecal microbiomes across the three cohorts were dominated by *Firmicutes* (62%) followed by *Bacteroidetes* (24%), *Actinobacteria* (5.2%) and *Proteobacteria* (4.2%) (Fig. 2). Bacterial members from *Verrucomicrobia*, *Tenericutes* and *Fusobacteria* were also detected in most of the subjects, although their abundance was observed to be low (0.03 to 0.05%) (Fig. 2).

**Figure 3:**
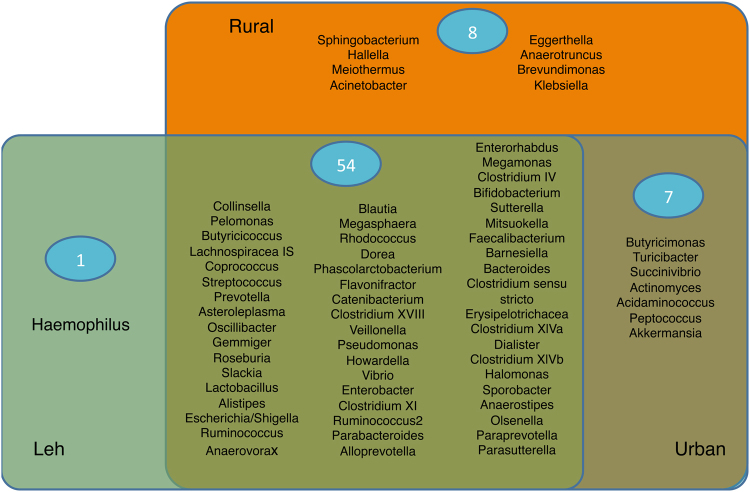
Venn diagram representing 54 core bacterial genera across the three cohorts. While the core microbiome from Leh was observed to be the least diverse (55 genera), the highest level of diversity was observed for those from the Ballabhgarh rural populations (62 genera).

In order to obtain a comparative picture of the core microbiome across the three cohorts, we obtained the sets of core genera (present in at least 50% of the samples with a median abundance of at least 0.01%), individually for ‘Leh’, ‘Ballabhgarh rural’ and ‘Ballabhgarh urban’ cohorts. A total of 70 genera were observed to constitute the core gut microbiome of at least one of the cohorts (Fig. 3). Notably, 54 (77%) were observed to be present in the core microbiome across all the three cohorts, indicating a high similarity in the core microbiome across the three regions. However, within the three regions, the core gut microbiome of the Leh population was observed to be the least diverse with 55 core genera. Individuals from Ballabhgarh rural region were found to have the highest numbers of core bacteria (Fig. 3).

**Figure 4:**
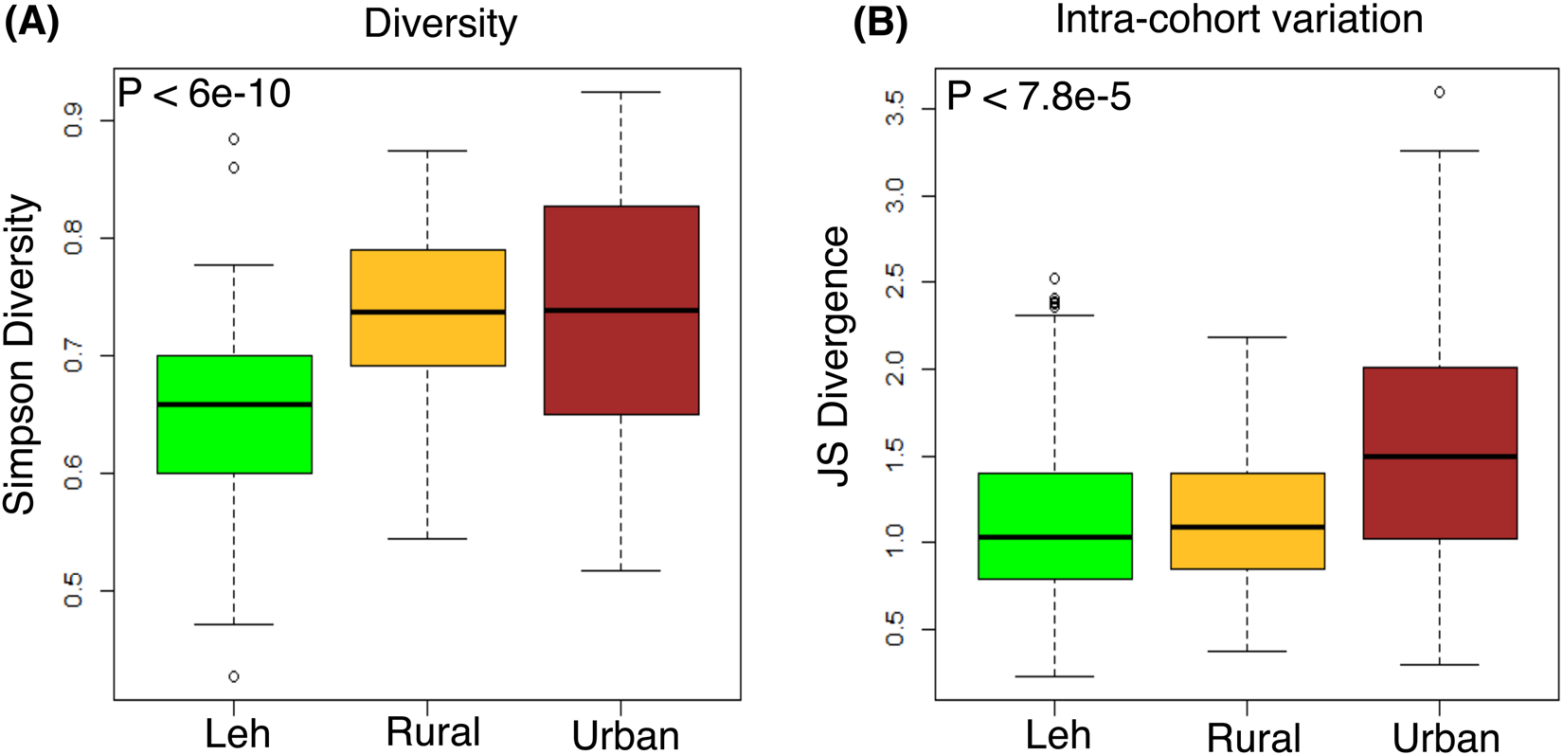
(**A**) Alpha diversity and (**B**) Intra-cohort Jensen-Shannon divergences of the gut microbiomes in the three cohorts. The gut microbiomes from Leh were observed to be the least diverse and highly homogenous amongst each other. The gut microbiota from the Ballabhgarh urban were observed to have high diversity and variation. On the other hand, the Ballabhgarh rural samples in spite of their high diversity were observed to be homogenous (low intra-cohort variation).

Complimentary analyses of the species level Simpson diversities and intra-cohort variations (using Jensen-Shannon divergence) in the species makeup of the gut microbiota displayed significantly distinct patterns pertaining to the overall gut microbial community structure across the three regions (Fig. 4). As observed for the core microbiome composition, the gut microbiome of Leh individuals were observed to have significantly lower species-level diversities as compared to those from Ballabhgarh urban and rural regions (P < 6e-10, Kruskal Wallis H test). The intra-cohort variation in the species-level make-up of the gut microbiota was also observed to be significantly lower, indicating homogeneity across individuals belonging to this cohort. In contrast, the individuals in the Ballabhgarh urban cohort were characterized by high diversity and high inter-individual variation in their gut microbiomes. The most interesting trend was observed for the Ballabhgarh rural cohort, where in spite of having a significantly high alpha diversity (as compared to the Leh region), the intra-cohort variations (beta diversity) in the gut microbial composition were significantly low as compared to those from the urban regions. In other words, gut microbiome of the Ballabhgarh rural individuals has a unique structure that is not only characterized by high diversity, but also a high degree of homogeneity within the same cohort.

### Variations in gut microbial community across regions

**Figure 5:**
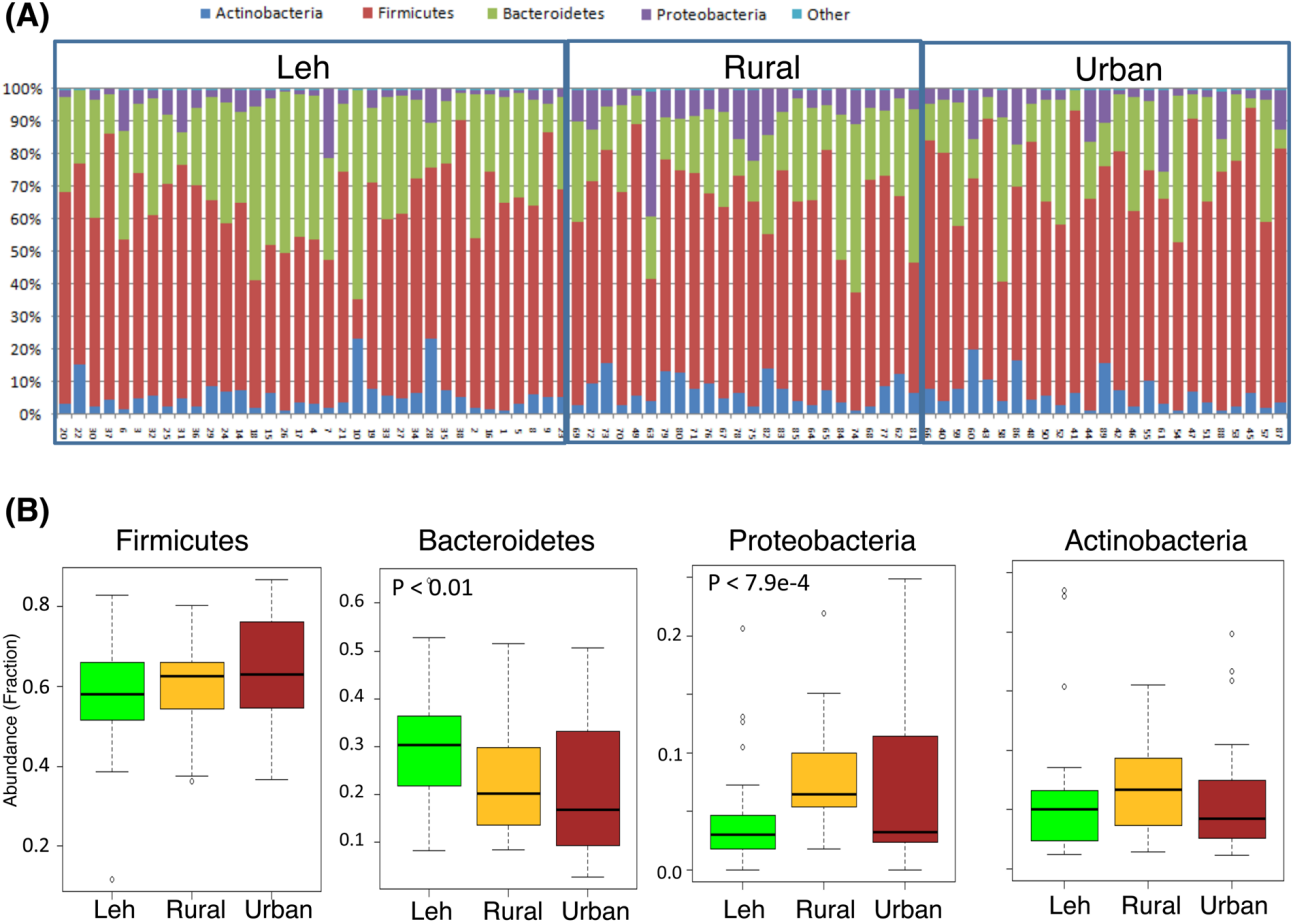
(**A**) Microbial diversity (Phylum level) of each subject living in three different areas. (**B**) Cumulative abundances of the four major bacterial phyla across the three cohorts. Compare to other two regions, subjects living in the Leh harbor higher numbers of *Bacteroidetes* and low numbers of *Proteobacteria*.

PERMANOVA analysis indicated region to have significant influence on the microbial community profiles at the phylum (P < 0.014), genus (P < 0.001) species (P < 4e-5) and OTU levels (P < 0.003) (Table 2). As compared to those from Ballabhgarh region, the gut microbiome of individuals from Leh were observed to have a distinct phylum signature characterized by a significantly higher abundance of *Bacteroidetes* (P < 0.012, Kruskal Wallis H test) and a markedly lower abundance of *Proteobacteria* (P < 0.0004, Kruskal Wallis H test) (Fig. 5).

**Table 2:** PERMANOVA analysis results of the association of microbial community structure at the levels of phylum, genus and species with region as the source of variation.

Taxonomic Level	Degree of Freedom (Df)	F	P value
Phylum	2	3.605	0.014
Genus	2	3.277	0.001
Species	2	3.416	0.001
Operational Taxonomic Unit (OTU)*	2	1.962	0.003

^*^Distances computed using Weighted Unifrac; Degree of Freedoms denotes the number of groups – 1; F-value is indicative of the ratio of the ‘within’ and ‘across’ group variances. The higher the F-value, the higher is the separation between the groups.

Random Forest classification (using 5-fold cross validation) indicated that, based on their genus level profiles, samples could be classified to their region-specific bins (i.e. Leh or Ballabhgarh) with an accuracy of 79% (Table 3). This indicated distinct genus level community signatures for the different regions. However, resolving the cohort affiliations further into ‘Ballabhgarh rural’, ‘Ballabhgarh urban’ and ‘Leh’ led to a noticeable decrease in the classification accuracy (accuracy of 60%) of Random Forest approach (Table 4). Examining the classification accuracies of the individual groups revealed that while the Leh and the Ballabhgarh rural gut microbiomes could still be distinguished with accuracies of 83% and 72% respectively, the classification accuracy for the Ballabhgarh urban cohort was extremely low (12.5%). This indicates that the gut microbiomes of Ballabhgarh urban individuals are extremely variable.

**Table 3:** Classification matrix of Random Forest based classification of the genus level microbial community profiles into (A) Leh v/s Ballabhgarh regions.

Actual Classification	Classified as	
	Leh	Ballabhgarh
Leh	27	8
Ballabhgarh	10	39

The rows indicate the actual affiliations. The columns indicate the predicted affiliations.

**Table 4:** Classification matrix of Random Forest based classification of the genus level microbial community profiles into Leh v/s Ballabhgarh rural v/s Ballabhgarh urban regions.

Actual Classification	Classified as		
	Leh	Ballabhgarh rural	Ballabhgarh urban
Leh	29	2	4
Ballabhgarh rural	6	18	1
Ballabhgarh urban	10	11	3

The rows indicate the actual affiliations. The columns indicate the predicted affiliations.

**Figure 6:**
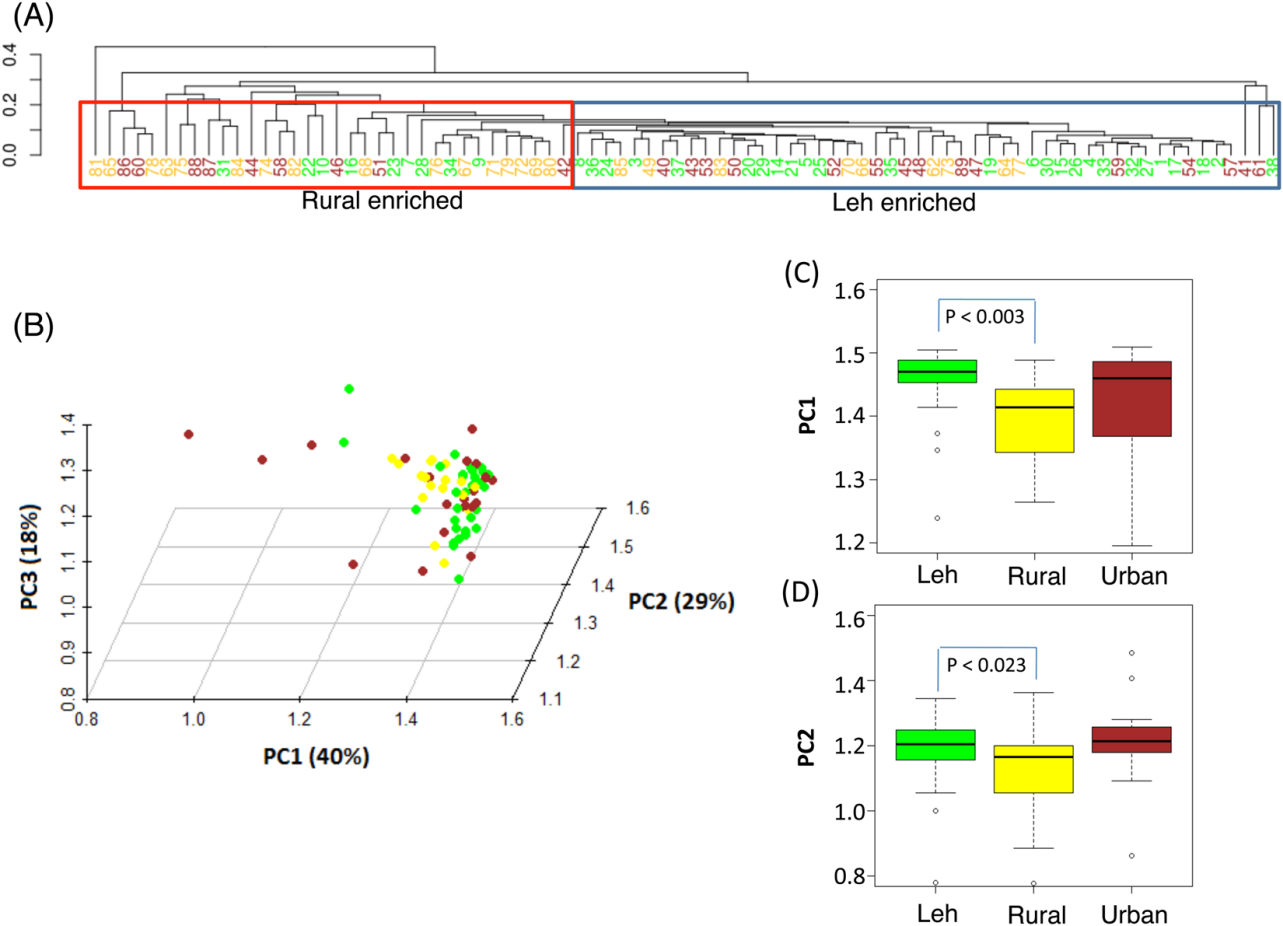
(**A**) Hierarchical clustering of the species-level profiles of the gut microbiomes. Samples from Leh, Ballabhgarh rural and Ballabhgarh urban populations are labeled in green, dark yellow and brown color, respectively. (**B**) Principal Coordinate Analysis plot of the gut microbiomes. Samples from Leh, Ballabhgarh rural and Ballabhgarh urban populations are labeled in green, light yellow and brown color, respectively. (**C**,**D**): Variation in the PC1 (**C**) and PC2 (**D**) components of the samples from the three cohorts. Overall, the trends indicate that while the gut microbiomes from Leh and Ballabhgarh rural regions have distinct community composition of their gut microbiomes, those from Ballabhgarh urban regions are observed to be variable.

This was further reflected at the species level profile. Hierarchical clustering of the species profiles revealed two groups, one enriched with the gut microbiome from Leh individuals and the other enriched for those obtained from the Ballabhgarh rural individuals. However, individuals from the Ballabhgarh urban population were observed to be spreaded across both the clusters (Fig. 6A). Furthermore, Principal Coordinate Analysis (PCoA) of the species level profiles elucidated that the Leh and Ballabhgarh rural gut microbiomes were observed to occupy distinct regions in terms of their Principal Component 1 (PC1) and Principal Component 2 (PC2) (Fig. 6B–D). However, no such signature could be observed for the Ballabhgarh urban samples indicating high variability.

### Region-specific signatures in gut microbial composition

**Figure 7:**
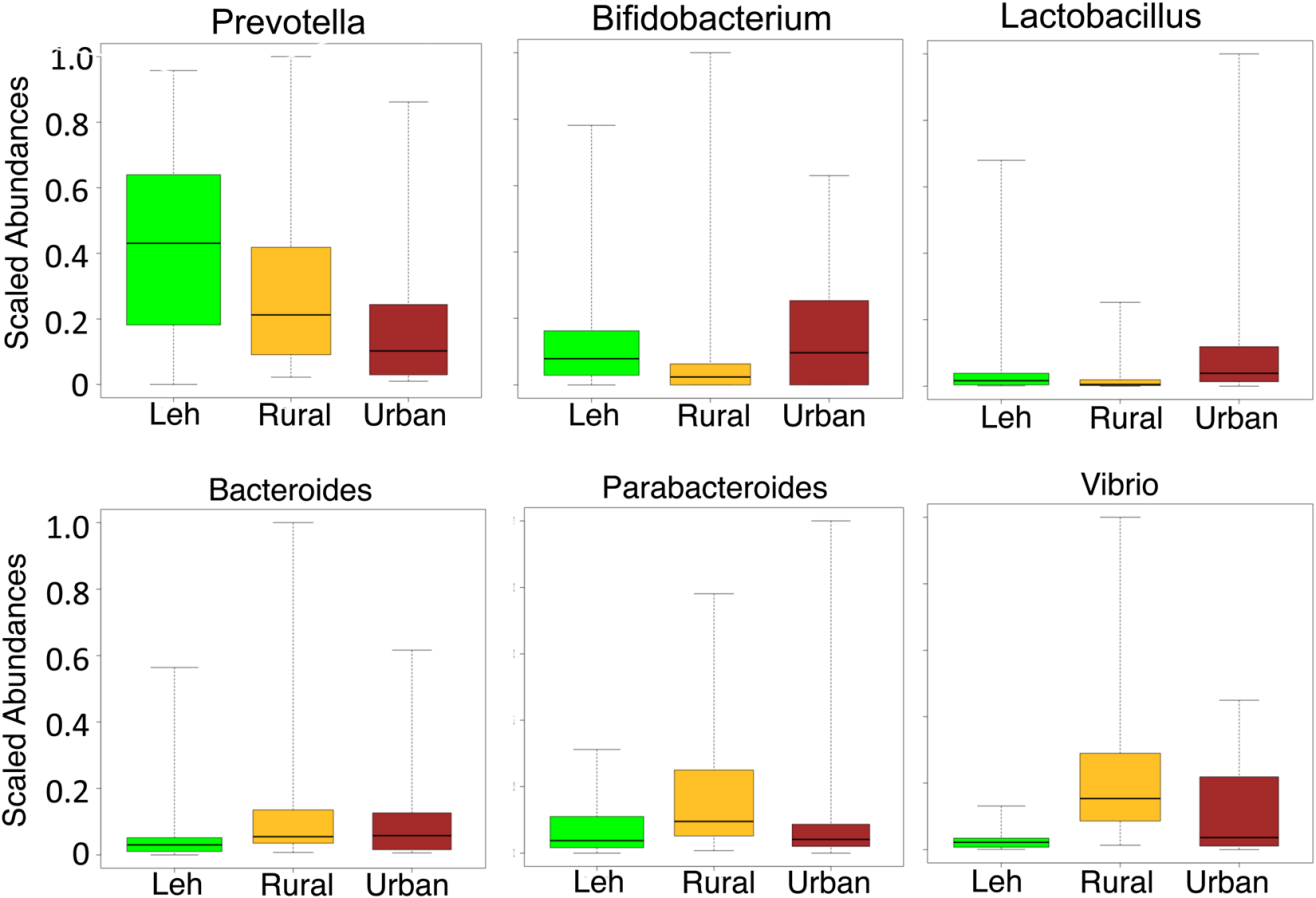
Variation of the scaled abundances of some of the prominent significantly different genera across the three regions. Please refer to the methods section for the methodology adopted for calculating the scaled abundances.

A total of 17 genera were observed to have significantly different abundance patterns across the three cohorts (Kruskal Wallis H P-value < 0.05, Benjamini Hochberg corrected P-value < 0.15) (Suppl. Table S3; Suppl. Fig. S1). Overall, certain genera or groups of genera were observed to show distinct behaviors across cohorts. *Prevotella* was observed to have the highest abundance in the Leh cohort, followed by Ballabhgarh rural and Ballabhgarh urban populations (Fig. 7). In contrast, *Parabacteroides*, *Blautia*, *Brevundimonas*, *Pelomonas* and *Megamonas* were observed to be significantly high in the Ballabhgarh rural population (Fig. 7, Suppl. Figs S1, S2A). Similarly, *Bacteroides*, *Vibrio*, *Eggerthela* and *Pseudomonas* were observed to be specific for the Ballabhgarh region, high in both the rural and urban populations (Fig. 7, Suppl. Fig. S1). *Lactobacillus*, on the other hand was selectively high in the Ballabhgarh urban population. Interestingly, there were some genera, namely *Bifidobacterium*, *Sporobacter* and *Gemmiger*, which were high in both Leh and the Ballabhgarh urban population, but significantly low in the Ballabhgarh rural population (Fig. 7, Suppl. Fig. S1).

To identify the region-specific species signatures, a similar investigation was performed at the species level. Region-specific species were identified using a combination of two criteria, that is, significant differences in the three cohorts (Kruskal Wallis H test with Benjamini Hochberg Corrected P-value < 0.10) and has high classification power for the individual cohorts using the Random Forest classifier (Mean decrease in accuracy of classification after removal of the species >0.5%). A total of 24 species were identified (as the set of region-specific species) that satisfied both the above criteria (Suppl. Fig. S2B). A majority of these species detected as specific to the Ballabhgarh rural region. These included *Vibrio cholerae*, *Vibrio albensis*, *Phascolarctobacterium faecium*, *Bacteroides salanitronis*, *Meiothermus silvanus*, *Pelomonas aquatica*, *Bacteroides intestinalis*, *Bacteroides fragilis*, *Bacteroides cellulosilyticus*, *Alistipes* sp. AP11, *Streptococcus thermophilus* as well as uncharacterized species of *Vibrio* and *Erwinia*. On the other hand, *Prevotella copri* and two uncharacterized species belonging to *Faecalibacterium* and *Lachnospiraceae* were observed to be highly abundant in the Leh population. As observed earlier, an uncharacterized species of *Lactobacillus* was observed to be specifically present in the Ballabhgarh urban population.

### Association of dietary patterns and composition of microbiota

The region-specific variations in the gut microbiome profiles could be due to the environmental factors as well as due to the diet. For this purpose, association (if any) of the dietary habits (Suppl. Table S2) of the individuals with the abundance of various genera was subsequently probed. Both PERMANOVA and ANOSIM analysis indicated that cooking oil and the diet (vegetarian v/s non-vegetarian v/s eggetarian) to have marginally significant effects on the genus level composition on the gut microbiomes (P-value < 0.099; Table 5).

**Table 5:** P-values of significance for the influence of various dietary habits on the gut microbiota composition obtained using PERMANOVA and ANOSIM.

Dietary Habit	ANOSIM P-value	PERMANOVA P-value
Cooking Oil	0.001	0.005
Eating Habit (Rice/Wheat/Both)	0.984	0.719
Sweet Tea Consumption	0.983	0.104
Non-Veg/Veg/Egg Diet	0.029	0.099
Dairy Intake	0.001	0.275
Fiber Intake	0.656	0.450

**Figure 8:**
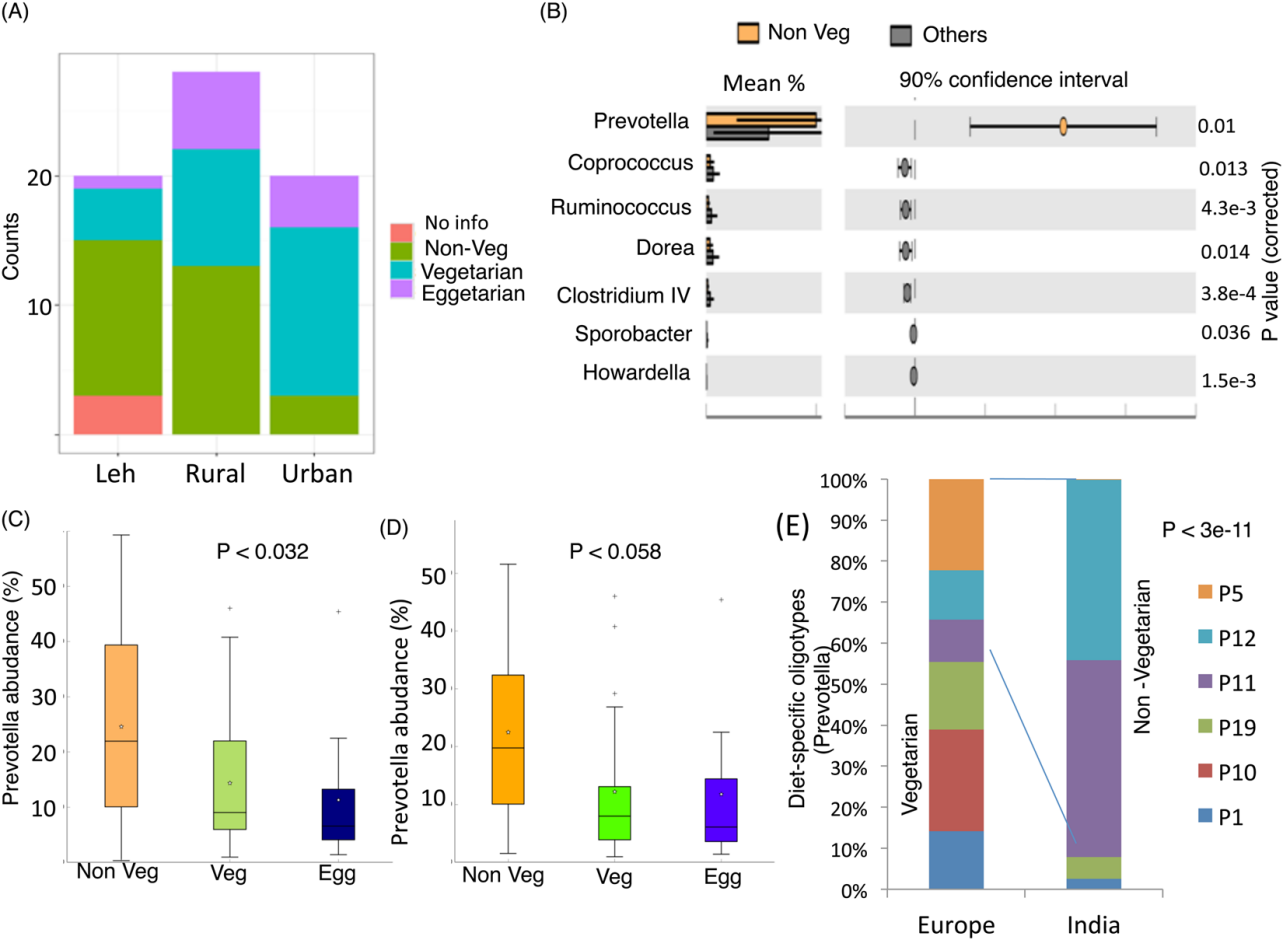
(**A**) Number of individuals with various dietary habits (Non-vegetarian/Vegetarian/Eggeterian) across the three cohorts. (**B**) Genera showing significant differences in the gut microbiomes of individuals having a non-vegetarian diet as compared to others (**C**) Variation of abundances of *Prevotella* in the gut microbiomes with the kind of diet of individuals in the Leh and Ballabhgarh regions. (**D**) Variation of abundances of *Prevotella* in the gut microbiomes with the kind of diet of individuals only in the Ballabhgarh regions. (**E**) Relative representation of diet-specific oligotypes of *Prevotella* in the European and Indian population.

While the Leh population was observed to have the highest percentage of individuals having non-vegetarian diet and individuals of the Ballabhgarh urban region were mostly observed to have vegetarian or eggetarian diet (Fig. 8A). Investigating the association of specific genera with dietary patterns identified seven genera, namely *Prevotella*, *Coprococcus*, *Clostridium*, *Ruminococcus*, *Howardella*, *Erysopelotrichaceae* and *Peptococcus*, that had significant differences in the abundances across the different dietary groups (Benjamini Hochberg corrected P-value < 0.05) (Suppl. Table S4). Of these, while *Prevotella* was observed to be specifically dominant in the non-vegetarian diet population, the rest were observed to have higher dominance in the vegetarian or eggetarian diet groups (Fig. 8B). The higher abundance of *Prevotella* in the non-vegetarian individuals was interesting (Fig. 8C), as it has been traditionally shown to be associated with individuals having a fiber-rich vegetarian diet^23^. A likely confounding factor for this trend could be the location (rather than dietary habits), as individuals from Leh were observed to have specifically higher abundance of *Prevotella*. To remove the possibility of location acting as a confounding factor, a comparison of *Prevotella* abundances in the various dietary categories was then performed only within Ballabhgarh population. Even within these populations, *Prevotella* was observed to be noticeably high (P < 0.037) in the gut microbiomes of non-vegetarian individuals (Fig. 8D). This indicated that the association of *Prevotella* with non-vegetarian diet (in Indians) was location independent.

A recent study^24^ had identified that, even within the genera *Prevotella* and *Bacteroides*, there were the presence of distinct oligotypes (synonymous with species sub-types) that had differential associations with vegetarian and omnivorous diets. In order to check the specific association of *Prevotella* with any of these oligotypes, the representative sequences of OTUs assigned to *Prevotella* were mapped onto the sequences representing each of the oligotypes identified in their study. Only 7% of the *Prevotella* OTU sequences could be fully mapped onto the known oligotype representatives, indicating that the *Prevotella* of the Indian population was unique as compared to those in the European population. Further, even within the known oligotypes (of the European population), the representation of the omnivorous (non-vegetarian) oligotypes was observed to be significantly high in the Indian individuals as compared to the European counterparts (Fig. 8E).

**Figure 9:**
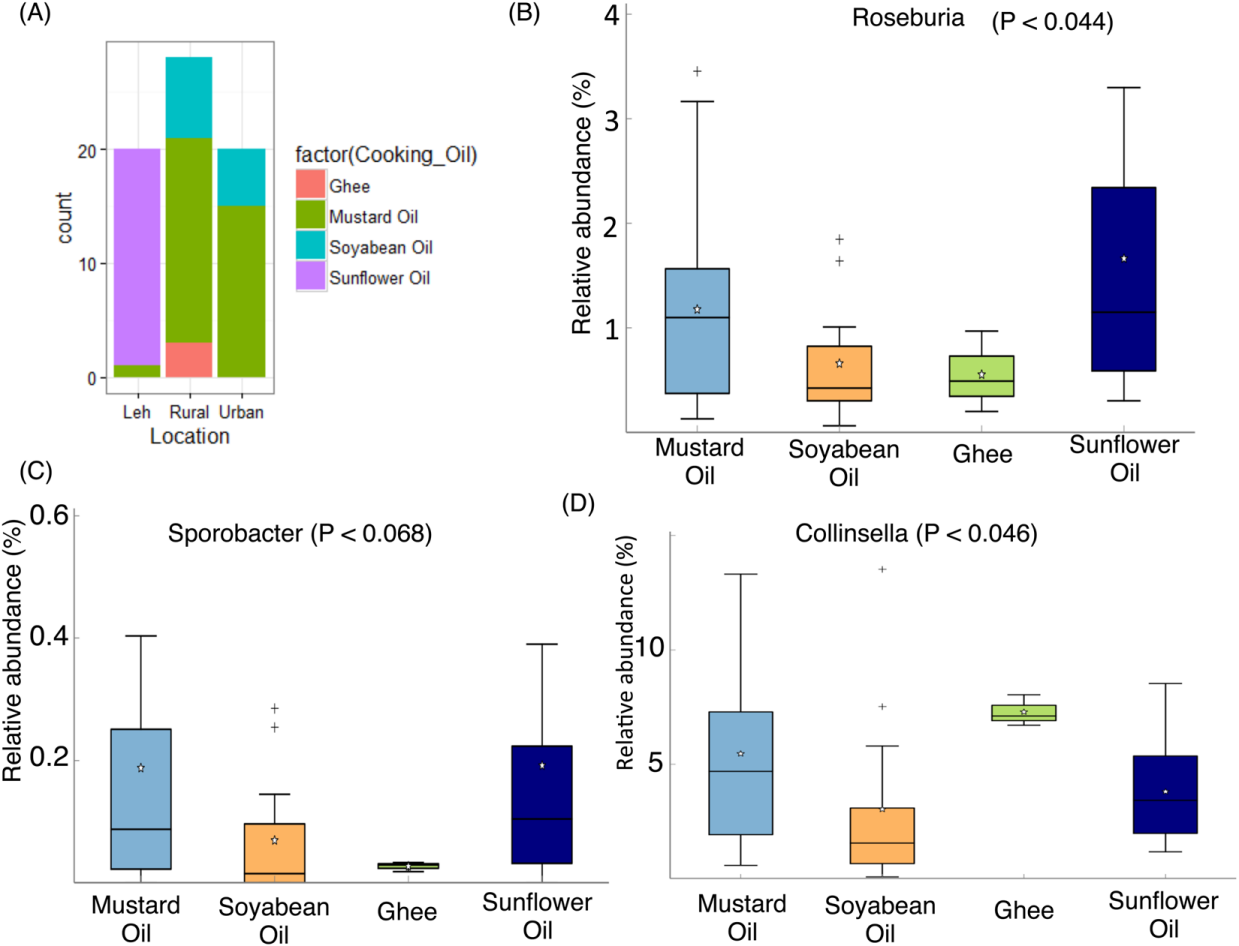
Cooking oil usage pattern of individuals across the three cohorts. (**B**–**D**) Variation of *Roseburia*, *Sporobacter* and *Collinsella* in the gut microbiomes of individuals using various cooking oils across the three cohorts.

Associations were also obtained for the different gut genera with the cooking oil being used (Suppl. Table S5). While sunflower oil seemed to be specifically used in the Leh population, the Ballabhgarh individuals seemed to be characterized by the use of mustard oil, soya bean oil and ghee (clarified butter) (Fig. 9A). Three kinds of components are generally used to describe the nutritional make up of cooking oils, namely poly-unsaturated fatty acids (PUFA), monounsaturated fatty acids (MUFA) and saturated fatty acids (SFA). Sunflower oil is known to have a high content of PUFA. Species belonging to *Roseburia* have been shown to have the degrading capabilities for PUFA, especially linoleic acids, in our gut microbiome^25^. In our study, a similar pattern was observed where in, *Roseburia* was observed to have the highest abundance in the group of individuals with sunflower oil consumption (Fig. 9B). A similar example was of *Sporobacter*, which has also been previously shown to be positively associated with PUFA consumption (Fig. 9C)^26^. In contrast, *Collinsella* was observed to be specifically abundant in the gut microbiome of individuals consuming ghee (Fig. 9D).

Furthermore, the Leh population was characterized by lack of dairy intake (Suppl. Fig. S3). This could be a probable reason for the absence of several genera like *Pseudomonas*, which have known to be associated with the dairy products^27^.

### Region specific trends in functional profile

Previous studies have shown that for healthy individuals in spite of differences at the taxonomic level the gut microbiomes show remarkably similar profile in functional repertoires. Consequently, to investigate the above aspect for the current microbiome datasets and to identify region-specific functional profiles (if any), we attempted to investigate the region-specific trends in the functional make-up of the gut microbiomes from the three different cohorts. Using microbial diversity obtained from the 16S rRNA gene amplicon data, we inferred functional profiles of the gut microbiomes by PICRUSt method^19^. To test the reliability of the predictions obtained using PICRUSt, we sequenced subset (n = 6) of the gut microbiomes from each cohort using shotgun-sequencing approach and compared the actual functional profiles, with that predicted using the PICRUSt approach. The predicted profiles for this subset of gut microbiomes obtained using PICRUSt were correlated with the actual functional profiles obtained as described. The predicted profiles showed a high degree of correlation with the actual functional profiles (R = 0.82, s.d = 0.3), indicating a high degree of accuracy of the PICRUSt based functional predictions of core gene pool.

**Figure 10:**
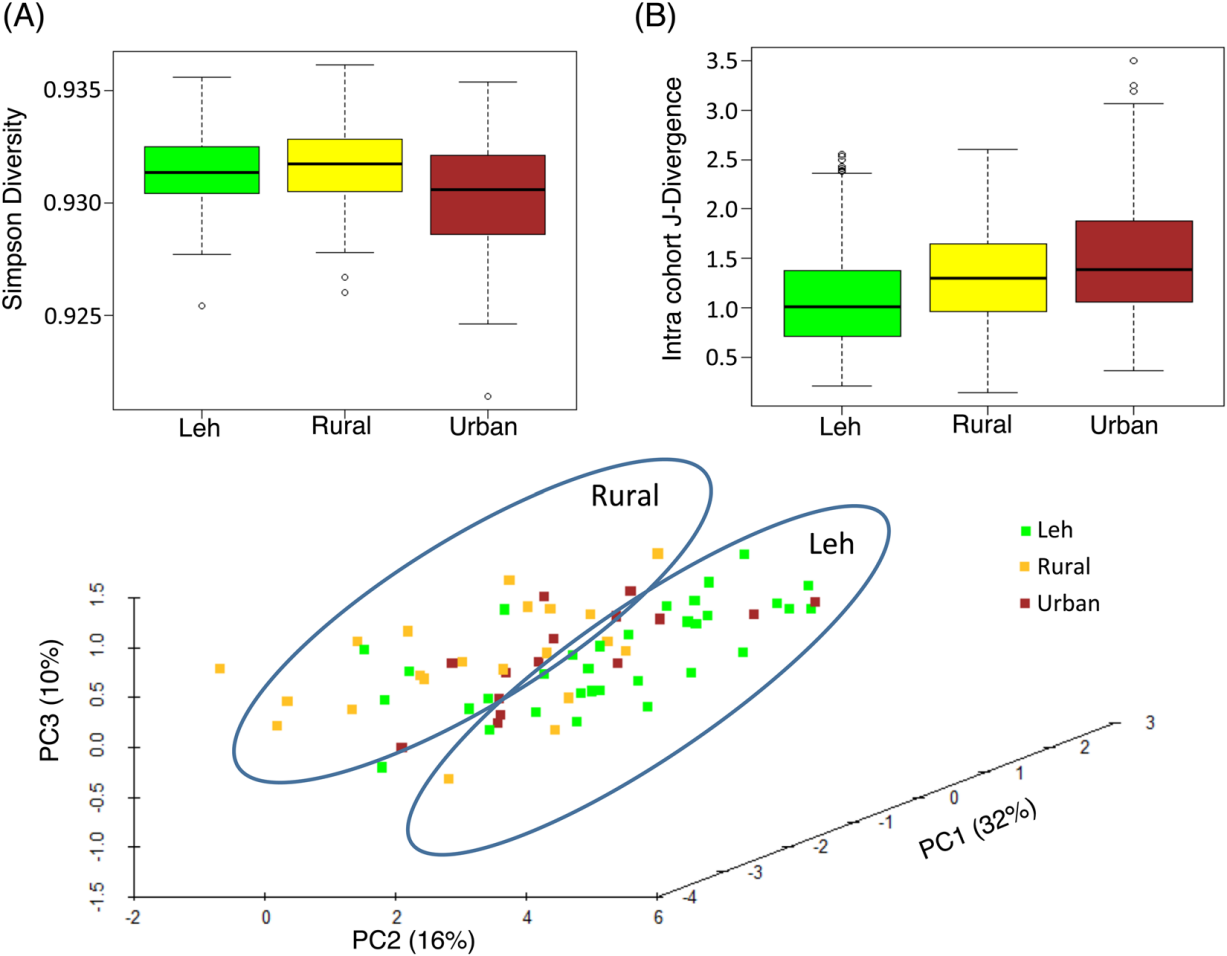
(**A**) Simpson Diversity and (**B**) Intra-cohort J-divergence of the process level functional profiles across the three cohorts. (**C**) Principal coordinate analysis plot showing the individual samples belonging to the three cohorts. The Leh and Ballabhgarh rural specific regions of the plot are indicated. Ballabhgarh rural and urban populations are indicated as ‘rural’ and ‘urban’ respectively.

In contrast to the observations made by previous studies, different cohorts were observed to have distinct trends in their functional profiles of the gut microbiomes (Fig. 10) (PERMANOVA F = 5.983, P < 0.002). These region-specific differences were observed both at the level of functional diversity (Fig. 10A) (P < 0.08) as well as for intra-cohort variations in the abundances of various functional processes (Fig. 10B) (P < 2.2e-16). While the predicted functional diversity of the gut microbiome from Leh and Ballabhgarh rural populations was observed to be similar, those of the Ballabhgarh urban populations were observed to be noticeably lower. Similarly, the intra-cohort variation within the gut microbiome of the Leh population was the lowest. In contrast, within Ballabhgarh, distinct trends were observed between the rural and urban populations, with rural individuals having significantly lower intra-cohort variation as compared to those from the urban cohort. This further indicates a high degree of gut microbial homogeneity within the individuals from Leh and a high variation in the functional profiles of the gut microbiome from the Ballabhgarh urban populations.

Principal Coordinate analyses revealed that, in general, the gut microbiomes from Leh and the rural Ballabhgarh were distinct in their functional profiles (Fig. 10C). On the other hand, the functional compositions of those belonging to the Ballabhgarh urban cohort were variable. These differences could be reflection of the exposure of individuals belonging to different environmental conditions and life-style habits.

**Figure 11:**
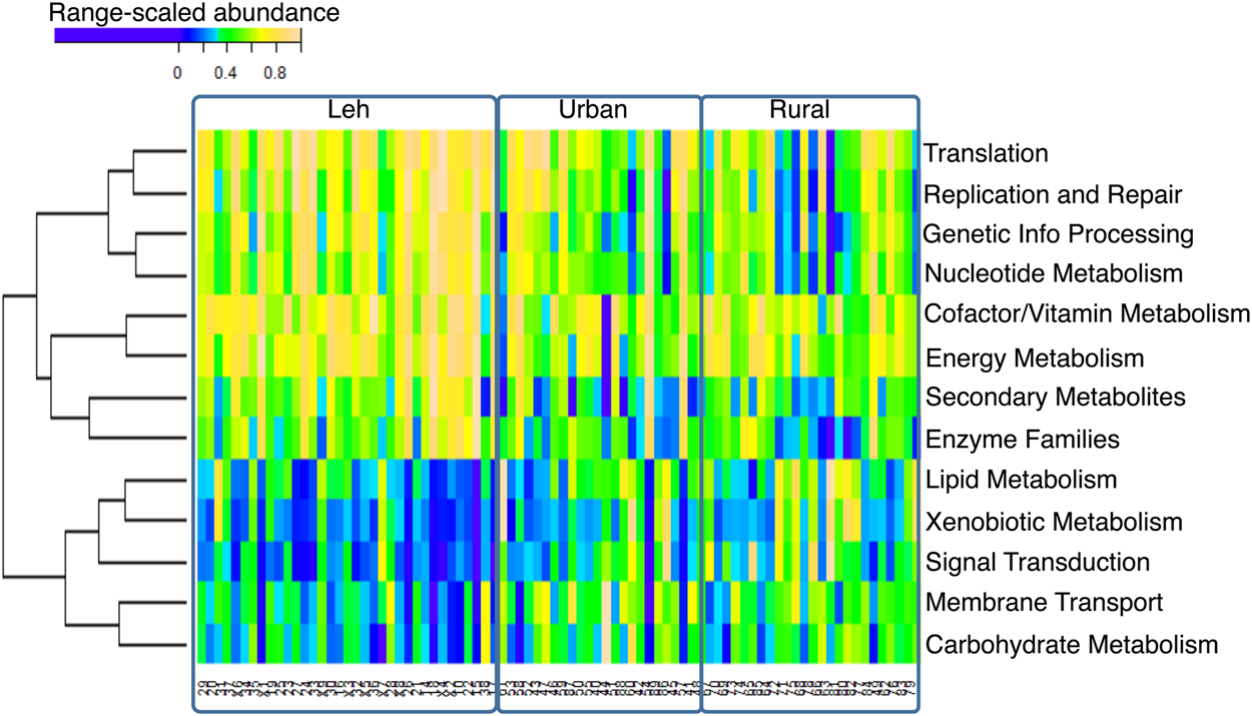
Range-scaled abundances of the significantly different processes across the three cohorts.

In order to investigate this aspect, we compared the abundances of specific functional processes and pathways across the three cohorts. The analysis identified a set of 13 different functional processes (Kruskal Wallis H test P – value < 0.05, FDR corrected using Benjamini Hochberg) (Fig. 11). It was observed that while several house-keeping processes like translation, DNA replication, nucleotide metabolism, energy metabolism, synthesis of co-factors/vitamins and metabolites were enriched in the Leh population, processes like carbohydrate and lipid metabolism, membrane transport, signal transduction and most importantly xenobiotic metabolism was observed to be higher in the Ballabhgarh rural and urban population. The abundance of pathways like xenobiotic metabolism in the Ballabhgarh population could be a consequence of higher exposure to industrial chemicals, pesticides, fertilizers and other drugs.

A deeper investigation of the significantly different pathways identified several pathways related to xenobiotic degradation like styrene degradation, dioxin degradation, benzoate/amino-benzoate/chloro-benzoate degradation, chlorohexane degradation to be significantly high in the Ballabhgarh rural population (Suppl. Table S6; Suppl. Fig. S4). Interestingly, two pathways, namely terpenoid synthesis and zeatin biosynthesis, which were observed to be high in Leh, are known for their anti-inflammatory mechanisms.

## Discussion

Distal gut, one of the most metabolically active organ in the human body, provides several functions to process dietary fibers and respond to environmental signals largely by utilizing microbial functional repertoire and its metabolites^28^. With respect to their genomes, humans are almost identical and their DNA sequences are 99.5% similar to any other humans^29^. In contrast, the composition of the human gut microbiome is fairly unique and quite stable over the time in each individual living across geography. The human gut microbiota, which harbors more than 5 million unique genes play important role in different aspects of host metabolism and immune functions^30^. Several trans-ethnic microbiome studies have reported that the ethnic background is strongly linked with specific microbial signature and metabolic pathways^31^. The ethnic diversity in India is very high and environment in different regions of the country is very heterogeneous. Nevertheless, different ethnic groups have different dietary habits and living in dissimilar environmental conditions. However, our knowledge on the gut microbiome and genetic repertoire of the microbes residing in the distal gut of healthy adult Indians living in different parts of the country is limited. The limited numbers of Indian subjects investigated for microbiome study left several questions to answer. Precisely, the previous studies reported that the gut microbiome of the Indians are dominated by the phylum *Bacteroidetes*^11–13^. However, the dietary components of Indians are mostly of plant origin. As compared to the *Bacteroides*, the genomes of most of the bacterial species belonging to the phylum *Firmicutes* are enriched with CAZymes, a class of enzyme important for the digestion of complex dietary fiber^1^. Therefore, the dietary habits of Indians and the gut microbiome composition and microbial genomic repertoires do not correlate with the reported findings. The present study however indicates the expected link between the observed dietary trends among Indians and their gut microbial makeup. Our analysis of gut microbiome of 84 healthy adult Indians living in low altitude Haryana (Ballabhgarh urban and rural) and high altitude Ladakh (Leh) regions revealed that the microbiome of healthy Indians are dominated by *Firmicutes* (62%), followed by *Bacteroidetes* (24%), *Actinobacteria* (5.2%) and *Proteobacteria* (4.2%) with prevalent and dominant members of *Prevotella*, *Bacteroides*, *Faecalibacterium*, *Roseburia*, *Ruminococcus*, *Clostridium*, *Dorea*, *Eubacterium*, *Bifidobacterium*, *Collinsella*, *Alistipes* and *Enterobacteriaceae*. Members of the *Verrucomicrobia*, *Tenerecutes* and *Fusobacteriaare* also present in the gut of most of the subjects, although their abundance is low.

Phylum level comparison of the gut microbiomes of subjects living in the Leh region revealed that their gut microbiota is highly homogenous and harbor higher numbers of *Bacteroidetes* and lower number of *Proteobacteria* compared to the subjects living in Ballabhgarh regions. Further analysis revealed that even within the Ballabhgarh populations, the gut microbial community showed distinct trends in its structure and inter-individual variations. People living in the Ballabhgarh urban region harbor highly diverse gut microbiota and maximum numbers of *Proteobacterial* genera compared to the rest of the regions. The intra-cohort diversity was also observed to be very high in the subjects living in the urban region. Principal Coordinate Analysis (PcoA) of gut microbiome compositions showed dispersed microbial distribution in the urban guts. On the other hand, the Ballabhgarh rural samples, in spite of their high diversity, were observed to be relatively homogenous (low intra-cohort variation). Further analysis of the prevalence and abundance of some of the significantly different genera across the regions showed *Prevotella* to be dominant in the gut of subjects living in the Leh region, whereas the gut microbiomes of Ballabhgarh rural subjects had a high representation of different *Proteobacteria* members like *Vibrio* and *Pseudomonas*.

The diet of the Leh population are rich in animal protein, whereas the diet of the Ballabhgarh rural and urban subjects are dominated by plant origin components. The differences in the microbial diversity between Leh and Ballabhgarh populations can be related to these differences in dietary intakes. In contrast to the observations in some of the previous studies linking dietary habits and gut microbiome, our analysis revealed a cohort-independent association of *Prevotella* with a nonvegetarian dietary pattern. Further sub-typing of the *Prevotella* group revealed that oligotypes P11 and P12 had a higher representation in the guts of Indian subjects. Their specific association of these oligotypes with animal origin foods has already been reported^32^. We also observed the influence of usages of various categories of cooking oil in the abundance pattern of specific genera. For example, *Collinsella* abundance was observed to be significantly high in the gut of subjects using ghee as a cooking oil. Interestingly, *Collinsella* has been previously linked with high serum cholesterol^33^. More importantly, increase in abundance of *Collinsella* has also been reported in the guts of individuals with symptomatic atherosclerosis^34^. However, establishing *Collinsella* as a risk factor would require further investigation of this bacterial group at the genomic level, specifically with respect to the genes related to metabolism of dietary lipids. In a similar manner, *Roseburia* and *Sporobacter* abundance, which were previously shown to be associated with various linoleic acid degradation^25,26^ were observed to be positively correlated with the usages of mustard oil and sunflower oil across the cohorts.

The current study, for the first time, reported that the different members of *Firmicutes* dominate the gut of the healthy Indians living in rural high altitude, urban and rural sea level altitude areas. Genomic analysis revealed that most of these abundant genera are enriched with large number of CAZyme encoding genes. Highest microbial diversity was detected in the study subjects living in rural low altitude areas. Furthermore, compared to the gut microbiome of Leh subjects, people living in the Ballabhgarh region harbor large number of genes that are linked with the membrane transport, carbohydrate metabolism, lipid metabolism, ion-channels and signal transduction pathways (Fig. 11). Most importantly, the gut microbiomes from Ballabhgarh were predicted to have a high abundance of genes belonging to several xenobiotic degradation pathways. This could be the reflection of the high exposure of these individuals to industrial/agricultural chemicals like fertilizers, pesticides as compared to the subjects from the high-altitude regions of Leh, which is relatively remote from industrialization and agricultural pollution. Furthermore, the subjects from Leh were observed to not only have the least abundance of *Proteobacteria*, but also a significantly high abundance of bacteria like *Faecalibacterium* and *Lachnospiraceae* that encode anti-inflammatory functions. Functions linked with the vitamin biosynthesis, energy metabolism and anti-inflammatory pathways like Zeatin biosynthesis are significantly high in the gut microbiome of Leh subjects.

These observations emphasize the importance of numerous common environmental exposures in shaping gut microbial ecology. Moreover, the similarity in overall pattern of the community structure suggest that despite the large influence of cultural factors, the similarity among members of each population across the three populations studied are remarkable.

## Conclusions

In the present study, a detailed analysis of the gut microbiome of healthy Indians clearly reflects the Firmicutes dominated microbiota in the Indian subjects living in two distinct geographical locations. Further analysis revealed distinct microbial signatures in each region. The minimal representation of *Proteobacteria* in the gut of Leh population is very attractive and representing a potential source of healthy gut microbiota in fecal microbiome transplantation-based therapeutics. Although all three groups had healthy individuals, yet rural community from low altitude areas had a unique microbiome characterized not only by a higher diversity, but also a higher degree of homogeneity within the same cohort. The insights from the current findings thus have high translational value, considering the identification of ideal subjects as donor for fecal microbiome transplantation.

### Ethics approval and consent to participate

IEC/NP-28/09.01.2015,OP-2/01.04.2016

### Availability of data and materials

Sequence reads and metadata for all the subjects enrolled in this study have been deposited in MG-RAST database (http://metagenomics.anl.gov/mgmain.html?mgpage=mydata) under the project name “Comparative Metagenomics of Gut Microbiota of Rural and Urban Healthy Indian Communities in Low Altitude and High Altitude Areas”. The assigned job numbers for all the sequences are 310885 to 311024. Details of taxonomic profile, statistical analysis taxonomic classifications are provided in the additional files.

## Electronic supplementary material

Supplementary information

Supplementary Table S1

Supplementary Table S2

Supplementary Table S3

Supplementary Table S4

Supplementary Table S5

Supplementary Table S6
