# Supplementary material for: Analysis of the Gut Microbiome of Rural and Urban Healthy Indians Living in Sea Level and High Altitude Areas
Source: Sci Rep. 2018 Jul 4;8:10104. doi: 10.1038/s41598-018-28550-3 (PMC6031670; doi:10.1038/s41598-018-28550-3)
Supplement: Supplementary file 1 — Supplementary information [file 41598_2018_28550_MOESM1_ESM.pdf]

**Analysis of the Gut Microbiome of Rural and Urban Healthy Indians Living in Sea Level and High Altitude Areas**

Bhabatosh Das[1@], Tarini Shankar Ghosh[1], Saurabh Kedia[2], Ritika Rampal[2], Shruti Saxena[1], Satyabrata Bag[1], Ridhima Mitra[1], Mayanka Dayal[1], Ojasvi Mehta[1], Surendranath A[2], Simon PL Travis[3], Prabhanshu Tripathi[1], G. Balakrish Nair[1,4], Vineet Ahuja[2@]

## Supplementary Information

**Supplementary Tables:**

**Supplementary Table S1**: The average length, the number of sequences and the region affiliations of the 84 16S rRNA genes amplicon datasets analyzed in the current study.

**Supplementary Table S2**: Region specific that is, taxa showing significantly different region-specific trends, at (A) Genus and (B) Species level.

**Supplementary Table S3**: Dietary habits of the 69 individuals for whom the dietary data could be obtained across the three cohorts.

**Supplementary Table S4**: Statistical significance of the variation of the various gut genera in the individuals with different dietary habits (Vegetarian/Non-Vegetarian/Eggetarian). The mean relative abundances of each genus in the corresponding groups are also indicated.

**Supplementary Table S5**: Statistical significance of the differences in the abundance of various gut genera in individuals using various cooking oils. The mean relative

abundance of the genera in the gut microbiome of individuals of various cooking oil categories is also indicated.

**Supplementary Table S6:** Significantly different processes and pathways in the Leh and Ballabhgarh rural cohort.

**Supplementary Figures:**

**Supplementary Figure S1:** Variation of the scaled abundances of some of the other significantly different genera (besides the ones shown in Figure 6) across the three regions.

**Supplementary Figure S2:** Scaled abundances of the (A) Genera and (B) Species showing significant differences (within the three cohorts) across the 84 samples. In (B) region specific trends of the same species have been indicated.

**Supplementary Figure S3:** Dairy intake tends in the individuals belonging to the three cohorts.

**Supplementary Figure S4**: Functional pathways inferred to be significantly high ($P < 0.10$ in Benjamini-Hochberg corrections) in (A) Ballabhgarh rural and (B) Leh populations.

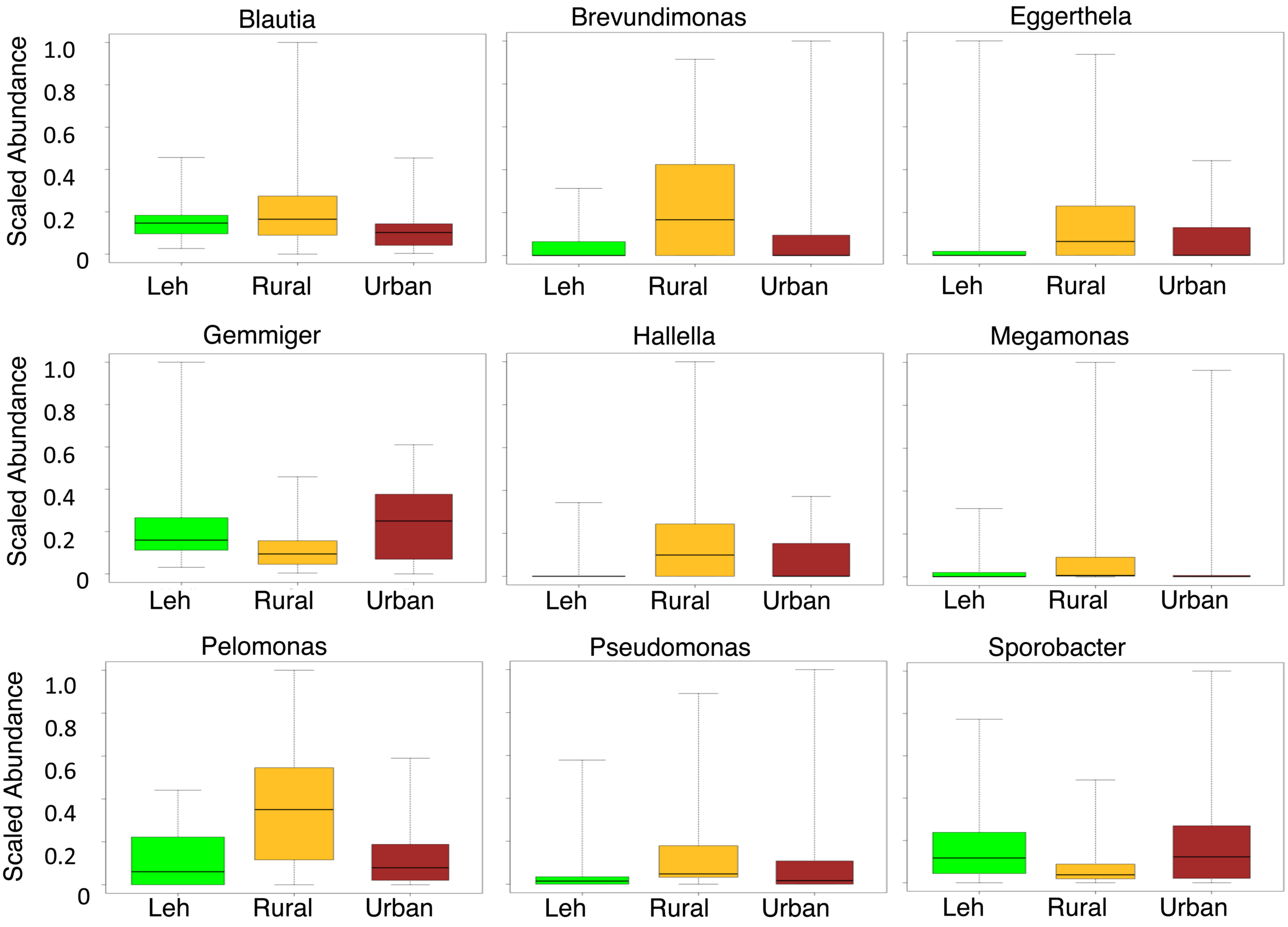


Suppl. Figure 1

**(A)**

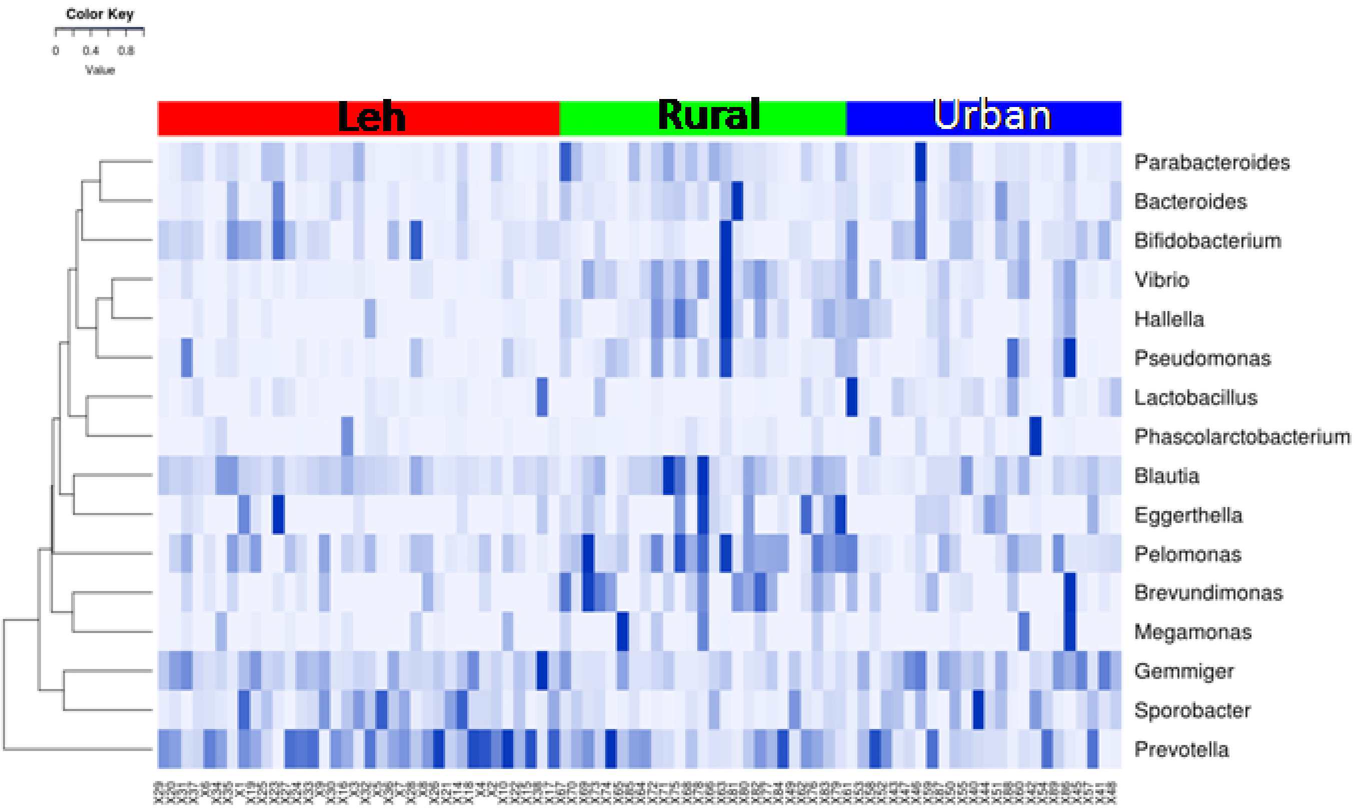


**(B)**

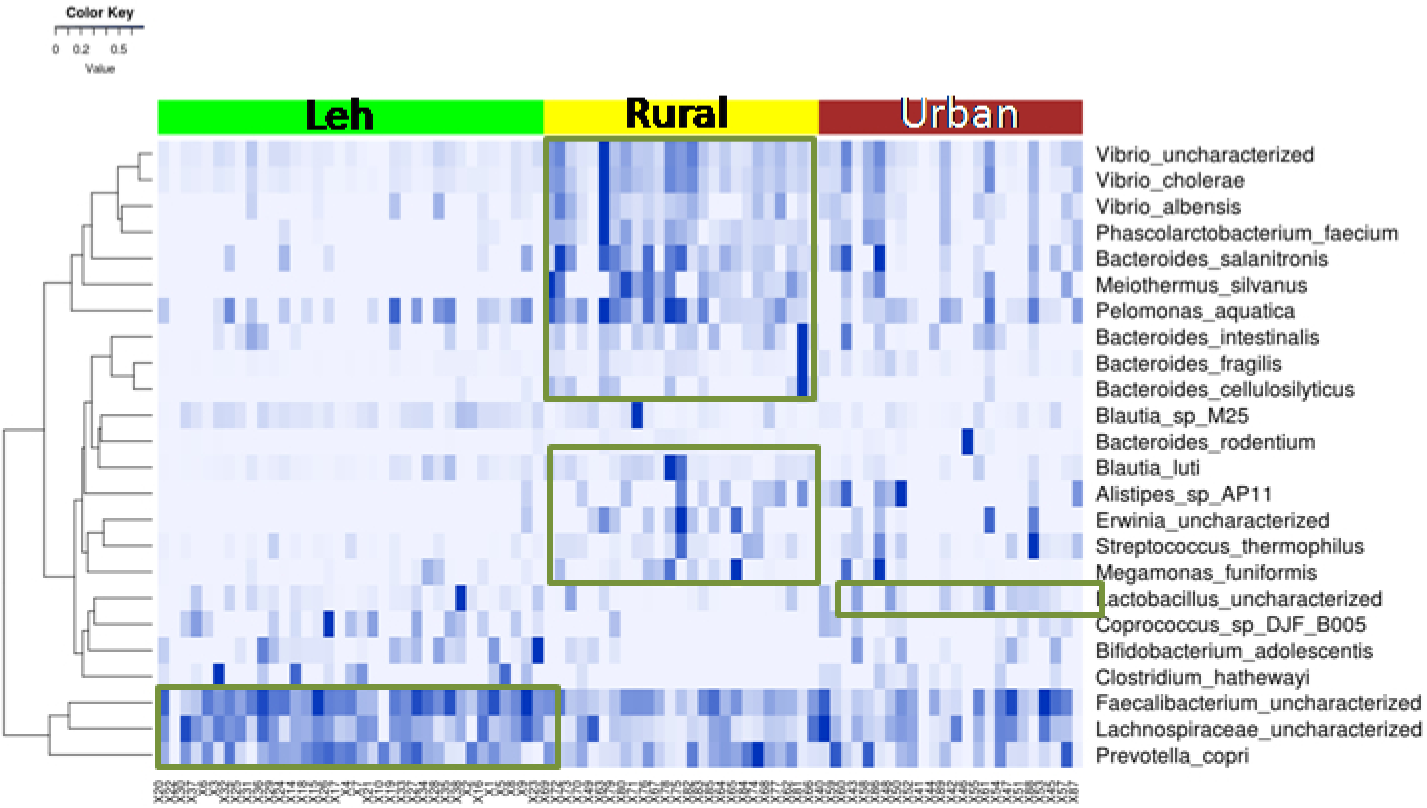


Suppl. Figure 2

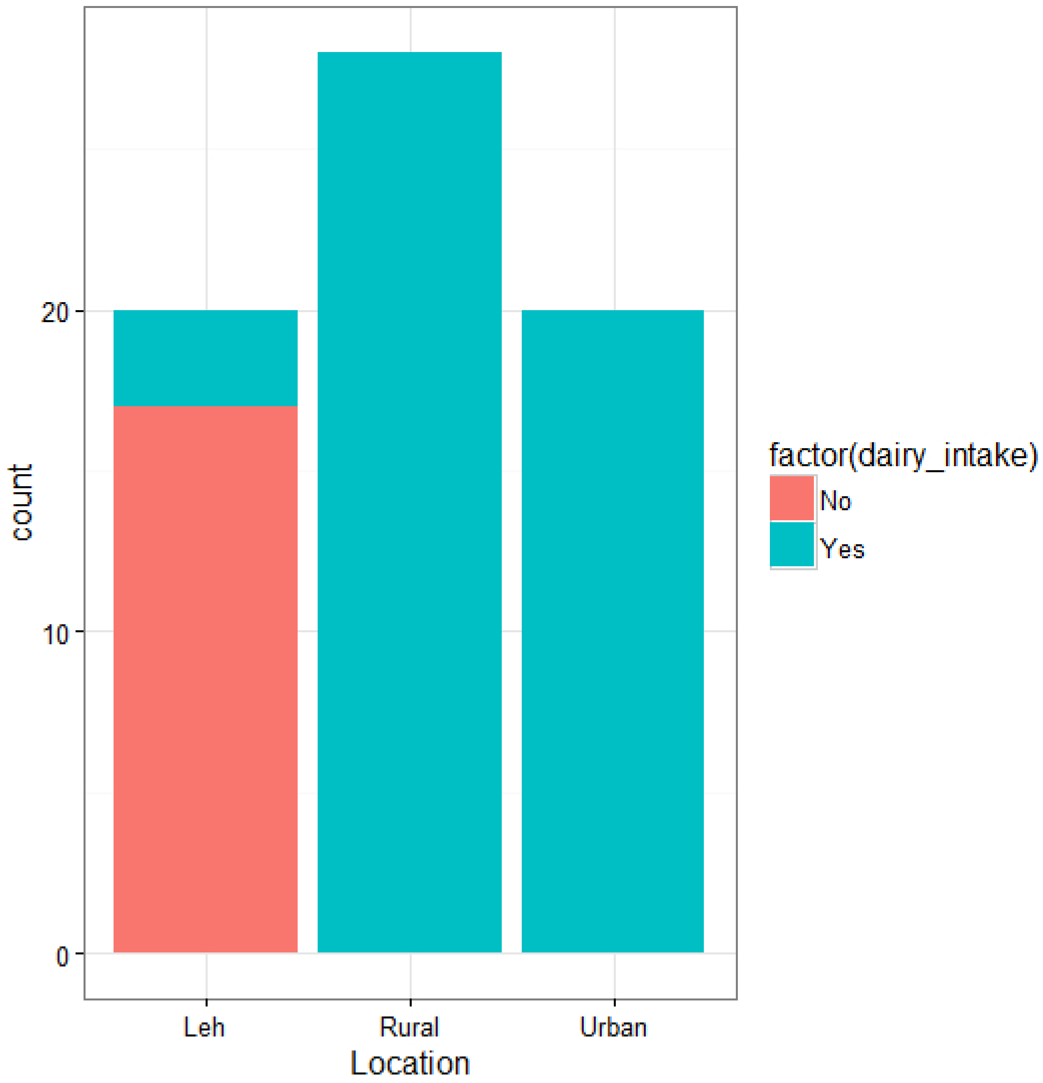


Suppl. Figure 3

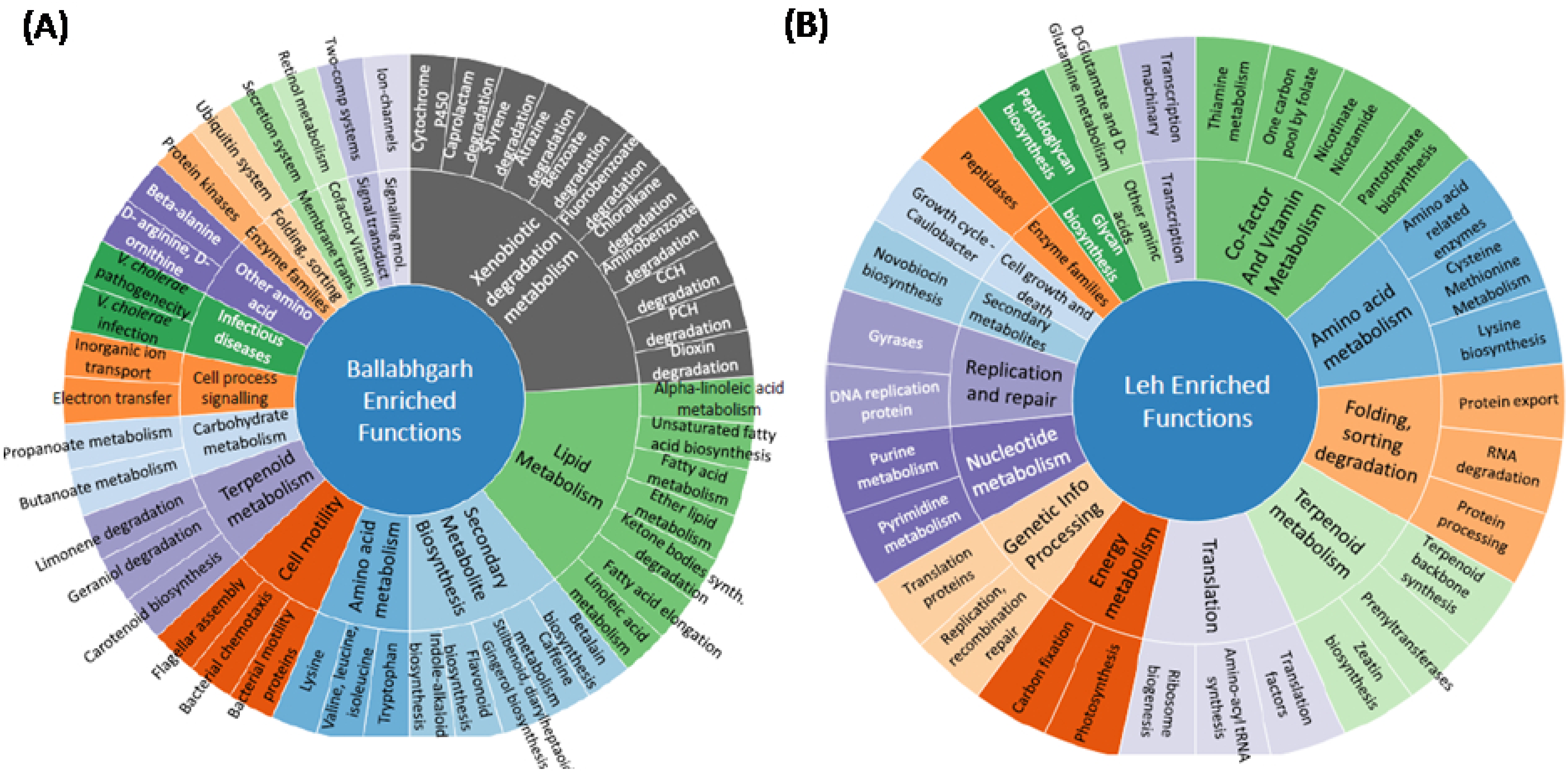


Suppl. Figure 4
